# Prevalence and Transmission of *Trypanosoma cruzi* in People of Rural Communities of the High Jungle of Northern Peru

**DOI:** 10.1371/journal.pntd.0003779

**Published:** 2015-05-22

**Authors:** Karen A. Alroy, Christine Huang, Robert H. Gilman, Victor R. Quispe-Machaca, Morgan A. Marks, Jenny Ancca-Juarez, Miranda Hillyard, Manuela Verastegui, Gerardo Sanchez, Lilia Cabrera, Elisa Vidal, Erica M. W. Billig, Vitaliano A. Cama, César Náquira, Caryn Bern, Michael Z. Levy, Working Group on Chagas Disease in Peru

**Affiliations:** 1 American Association for the Advancement of Science (AAAS) Science & Technology Policy Fellow at the Division of Environmental Biology, National Science Foundation, Arlington, Virginia, United States of America; 2 Bloomberg School of Public Health, Johns Hopkins University, Baltimore, Maryland, United States of America; 3 Department of Pediatrics and Department of Emergency Medicine, University of Arizona, Tucson, Arizona, United States of America; 4 Faculty of Science and Philosophy Alberto Cazorla Talleri, Urbanización Ingeniería, University Peruana Cayetano Heredia, Lima, Peru; 5 Center for Clinical Epidemiology and Biostatistics, Department of Biostatistics and Epidemiology, Perelman School of Medicine, University of Pennsylvania, Philadelphia, Pennsylvania, United States of America; 6 Centers for Disease Control and Prevention, Atlanta, Georgia, United States of America; 7 Department of Epidemiology and Biostatistics, School of Medicine, University of California, San Francisco, San Francisco, California, United States of America; Universidad Autónoma de Yucatán, MEXICO

## Abstract

**Background:**

Vector-borne transmission of *Trypanosoma cruzi* is seen exclusively in the Americas where an estimated 8 million people are infected with the parasite. Significant research in southern Peru has been conducted to understand *T*. *cruzi* infection and vector control, however, much less is known about the burden of infection and epidemiology in northern Peru.

**Methodology:**

A cross-sectional study was conducted to estimate the seroprevalence of *T*. *cruzi* infection in humans (n=611) and domestic animals [dogs (n=106) and guinea pigs (n=206)] in communities of Cutervo Province, Peru. Sampling and diagnostic strategies differed according to species. An entomological household study (n=208) was conducted to identify the triatomine burden and species composition, as well as the prevalence of *T*. *cruzi* in vectors. Electrocardiograms (EKG) were performed on a subset of participants (n=90 *T*. *cruzi* infected participants and 170 age and sex-matched controls). The seroprevalence of *T*. *cruzi* among humans, dogs, and guinea pigs was 14.9% (95% CI: 12.2 – 18.0%), 19.8% (95% CI: 12.7- 28.7%) and 3.3% (95% CI: 1.4 – 6.9%) respectively. In one community, the prevalence of *T*. *cruzi* infection was 17.2% (95% CI: 9.6 - 24.7%) among participants < 15 years, suggesting recent transmission. Increasing age, positive triatomines in a participant's house, and ownership of a *T*. *cruzi* positive guinea pig were independent correlates of *T*. *cruzi* infection. Only one species of triatomine was found, *Panstrongylus lignarius*, formerly *P*. *herreri*. Approximately forty percent (39.9%, 95% CI: 33.2 - 46.9%) of surveyed households were infested with this vector and 14.9% (95% CI: 10.4 - 20.5%) had at least one triatomine positive for *T*. *cruzi*. The cardiac abnormality of right bundle branch block was rare, but only identified in seropositive individuals.

**Conclusions:**

Our research documents a substantial prevalence of *T*. *cruzi* infection in Cutervo and highlights a need for greater attention and vector control efforts in northern Peru.

## Author Summary

Chagas disease causes significant morbidity and mortality throughout Central and South America. The epidemiology and control of this disease is subject to unique regional particularities, including the behavior and ecology of the local insect vector species. Significant resources have been allocated towards research and control efforts in southern Peru, yet very little is known about the prevalence and epidemiology of *Trypanosoma cruzi* in northern Peru. Our study highlights significant *T*. *cruzi* infection in northern Peru and is one of the first to document substantial transmission by the insect *Panstrongylus lignarius*. Our results illustrate major gaps in knowledge and the need for public health interventions targeted at Chagas disease in the region of Cutervo Province of northern Peru.

## Introduction

Chagas disease is caused by the protozoan parasite *Trypanosoma cruzi*, and is primarily transmitted by triatomine vectors. Chagas disease is endemic to poor rural regions of Central and South America and is responsible for the largest public health burden of any parasitic infection in the Western Hemisphere [1]. An estimated 8 million people are infected with *T*. *cruzi* and millions more are at risk [2]. *Trypanosoma cruzi* is carried in the gut of the triatomine vector and transmitted through the insect’s feces. While the vector-borne route predominates, oral transmission, congenital transmission and infection through blood transfusion and organ transplantation also occur.

Acute Chagas disease is asymptomatic or oligosymptomatic and if clinical manifests as fever and fatigue. The majority of individuals will survive this acute phase without treatment or even evaluation [2]. Approximately 20–30% of chronic infections advance to the chronic symptomatic form of the disease, characterized by cardiac, gastrointestinal or neurologic disease [2–4].

Heart disease is the most common clinical manifestation of chronic Chagas disease [2]. In Peru gastrointestinal and neurologic forms are extremely rare. Chagas heart disease is an irreversible fibrosing inflammatory cardiomyopathy characterized by conduction abnormalities, such as right bundle branch block, left anterior fascicular block, ventricular extra systoles and ventricular tachycardia [2]. As the disease progresses, manifestations include sinus node dysfunction, atrioventricular blocks, dilated cardiomyopathy and thromboemboli [2].

Chagas disease is understudied in northern Peru and little is known about the epidemiology of *T*. *cruzi* in the region [5]. *Panstrongylus lignarius* (synonymous with *Panstrongylus herreri*) [6] is known as the 'main domestic vector' of Chagas disease in northern Peru, specifically in the Marañon Valley, yet several other species have been described in northern Peru [7]. We conducted a series of cross-sectional surveys in several communities of Cutervo Province, in the Cajamarca region of Peru. The study aims were to (1) describe the seroprevalence of *T*. *cruzi* in humans, domestic dogs, and guinea pigs; (2) to describe the species and prevalence of vectors overall and with *T*. *cruzi;* (3) identify and characterize risk factors of *T*. *cruzi* infection in humans; and (4) characterize the extent and scope of cardiac abnormalities associated with *T*. *cruzi* infection in humans.

## Materials and Methods

### Study Area and Population

**Fig 1:**
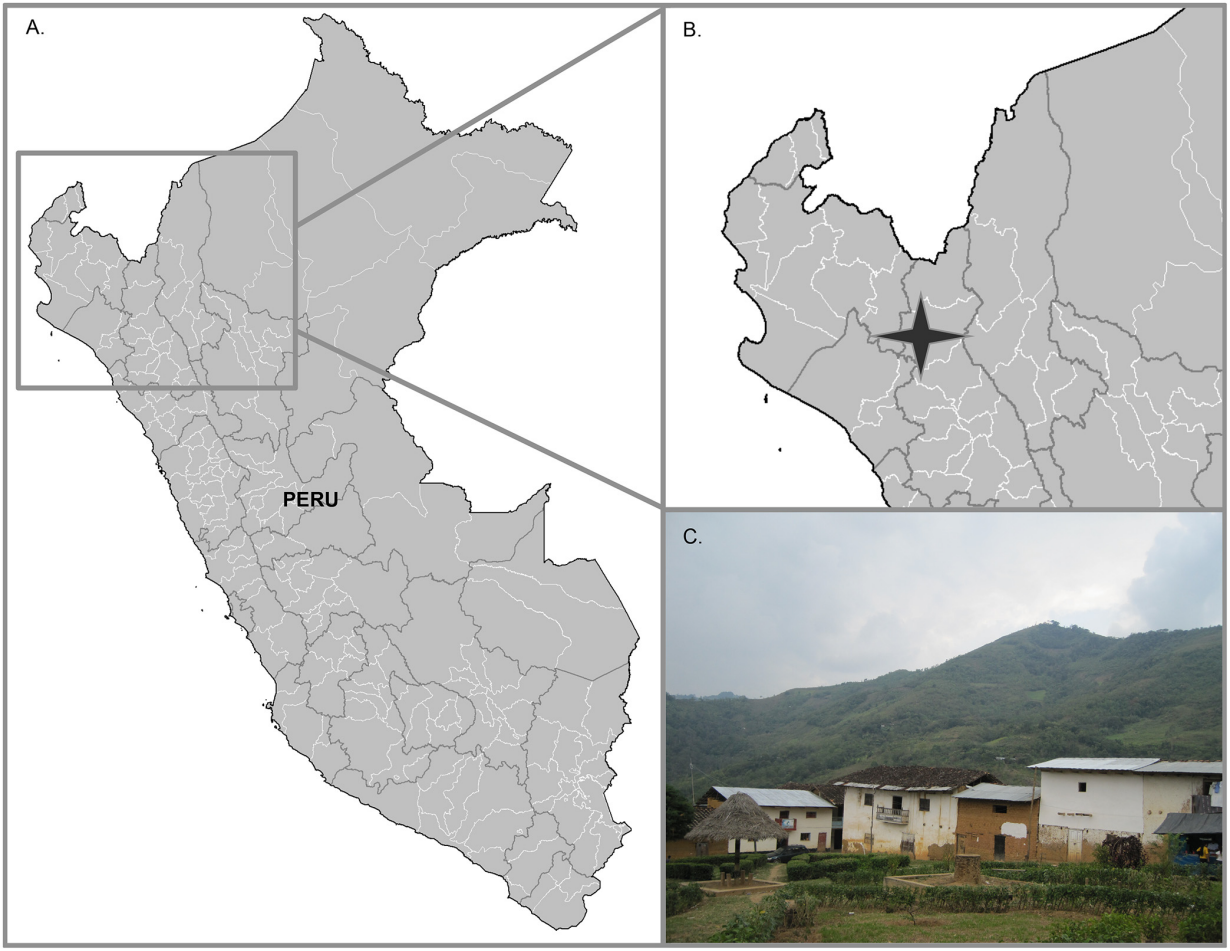
A map and photo depicting the study region in the Peruvian high jungle. A map of Peru shows the region that contains the Huancabamba River Valley (A) and an inset illustrates where the communities of Casa Blanca, La Esperanza, Campo Florido, Pindoc, Rumiaco and Nuevo Guayaquil are situated (B). A photograph illustrates the town center of Campo Florido to exemplify close proximity of houses, crude housing materials, and mountainous terrain (C). Photo: Alroy.

This study was conducted in December 2009 to October 2010, in Cutervo Province of Cajamarca, Peru. Cutervo is located in the Huancabamba River Valley, near the Marañon Valley of the Andes (altitude 850–1700 m), which ultimately drains into the Amazon River Basin (Fig 1). Six communities (Campo Florido, Casa Blanca, La Esperanza, Pindoc, Nuevo Guayaquil and Rumiaco) were included in the study based on government documented triatomine infestation and clinical reports of people with Chagas disease. All communities were located within an aerial distance of 15 km. They share the same ecoregion, known as the Peruvian Yungas or Selva Alta, which is characterized by neotropical forest, steep slopes and narrow valleys. Road infrastructure and access to these communities, however, was variable: Casa Blanca and La Esperanza were connected to the local highway via a gravel road; the community of Campo Florido, however, could only be reached by a poorly maintained dirt road that was impassable for several months during the rainy season. All six communities were included in the human serological survey and the electrocardiogram (EKG) study. A subset of four communities was sampled for domestic dog serology and for domiciliary and peridomestic vectors (Campo Florido, Casa Blanca, La Esperanza, and Pindoc) and one community (Campo Florido) was evaluated for guinea pig serology.

Trained study nurses recruited participants both at the local health posts during a community-wide serological testing campaign and at people’s homes during house-to-house visits.

### Ethics Statement

All study participants provided informed written consent and a parent or guardian provided written consent on behalf of minors. A fingerprint, as a proxy of a written signature, was an acceptable alternative for individuals unable to write. With participant consent, all human seropositive individuals were referred to the Ministry of Health. Animal owners provided written consent for the participation of domestic animals. The methods of this study complied with federal and institutional regulations. The Institutional Review Board (IRB) of the Asociación Benéfica Proyectos en Informática, Salud, Medicina y Agricultura (Lima, Peru) approved the protocol (file# CE0886.09) as did the IRB of the University of Pennsylvania (file# 812713). The animal protocol was reviewed and approved by the Institutional Animal Care and Use Committee of the Universidad Peruana Cayetano Heredia (UPCH) (file# 52186) as well as the University of Pennsylvania (file# 803364). The animal protocol adhered to standards outlined by the National Research Council's Guide for the Care and Use of Laboratory Animals [8].

### Human Study

All residents of the six communities >2 years of age were eligible to participate in the serological survey. The age and sex of both survey participants and non-participants were recorded. Blood samples were collected from each participant, stored at 4°C and were transported on the same day to the field laboratory. Blood was separated by centrifugation and stored at -20°C. Serologic analysis was completed at the Universidad Peruana Cayetano Heredia Laboratory of Infectious Diseases (LID-UPCH). All human serum specimens were tested by three assays: the Chagatek *T*. *cruzi* lysate ELISA (bioMerieux, Marcy l’Etoile, France), the Wiener Recombinant ELISA (Wiener, Rosario Argentina), and the trypomastigote excreted-secreted antigen (TESA) immunoblot [9]. *T*. *cruzi* infection in humans was considered confirmed if two or more tests yielded positive results [10]. Specimens with one or no tests positive were considered seronegative. The Chagatek and Wiener ELISA were completed according to manufacturer’s instructions and the threshold for positive results was 0.10 optical density (OD) units above the mean absorbance of two negative control specimens included on each plate. The TESA assay was completed according to specifications in Umezawa et al [9].

### Electrocardiogram Study

To understand the extent and scope of cardiac abnormalities in these communities and their association with chronic *T*. *cruzi* infection, an electrocardiographic study was conducted on 90 infected individuals and 170 controls. All participants of the serological survey were invited to the EKG study at the time of the serological survey recruitment. Controls were matched based on age and gender. A majority of infected individuals (80) were matched with two negative controls, and the remaining individuals (10) were matched with one. At the local health posts, participants underwent a structured medical history, a non-invasive physical exam (PE) by a study physician, and a 12-lead EKG in the 30° inclined position (portable Welch Allyn CP100). Parents were encouraged to be present for their children’s examinations. The duration of PEs and EKGs ranged from 15–30 minutes and all EKG data was subsequently read and coded by a board certified cardiologist. An EKG was considered to have abnormalities consistent with Chagas cardiomyopathy if one or more of the following were present: atrial fibrillation/flutter, junctional rhythm, ventricular tachycardia (sustained or non-sustained), ventricular extrasystoles (multiform, paired, or salvos), sinus node dysfunction, sinus bradycardia (<50 bpm), second degree AV block (type I or type II), third degree AV block, AV disassociation, left or right bundle branch block (LBBB, RBBB), left anterior or left posterior fascicular block, or trifascicular block [2,11,12]. Incomplete RBBB was not considered consistent with Chagas cardiomyopathy.

### Entomological Household Study

Four communities were evaluated in the household entomological survey: Campo Florido, Casa Blanca, La Esperanza, and Pindoc. With household member consent, two trained entomologic collectors, aided by a tetramethrin flushing-out agent (Sapolio, Mata Moscas), searched domestic and peridomiciliary habitats including domestic animal enclosures for a total of one half-hour (one person-hour). Captured triatomines were stored at 4°C until processing at the field laboratory and then examined for the presence of *T*. *cruzi*, following standard procedures [13,14]. Vector species was determined based on morphology. The species, quantity, sex and life stage of triatomine vectors was documented. Due to the specimen quality once the triatomines arrived at the field laboratory, not all of the collected triatomines were evaluated for sex, development stage, and intestinal contents. Second through fifth instar triatomines were evaluated for trypanosomatids. For each household the wall and roof construction material were documented; data on the total number and type of domestic animals were reported by the household representative.

### Domestic Animal Study

A serological survey of domestic animals was performed to document *T*. *cruzi* transmission through potential reservoir species. Domestic dogs (*Canis lupus familiaris*) from Campo Florido, Casa Blanca, La Esperanza, and Pindoc were evaluated, as were Guinea Pigs (*Cavia porcellus*) from Campo Florido. A household level census of all domestic species was conducted to estimate the domestic animal population. Canine age was reported by owners, and guinea pig age was approximated based on measured body length. Canine and guinea pig blood samples were collected by a veterinarian or trained phlebotomist, and, stray, pregnant, notably sick, and/or juvenile animals (dogs <1 mo, and guinea pigs < 20 cm in length) were not sampled. Transport and processing were identical to that of human blood samples, however, domestic animal serostatus was determined based on an enzyme-linked immunosorbent assay (ELISA). At LID-UPCH, the domestic animal sera were tested for the presence of anti *T*. *cruzi* antibodies by epimastigote alkaline extract (EAE) ELISA using Arequipa strain epimastigote extracts (2.5 ug/ mL) [15]. Each plate contained seven negative and one positive control. The positive control consisted of sera from either a Y strain experimentally infected guinea pig or from an Arequipa strain naturally infected dog. The sample was positive if the OD was greater than three standard deviations above the mean plate OD. A subset of canine and guinea pig samples (n = 103 and n = 31, respectively) was evaluated by TESA-blot [9].

### Analysis Methods

Descriptive statistics were first used to characterize the human study population and compare demographic information to the general population from which they were selected. The infection prevalence along with exact binomial 95% confidence intervals was ascertained for humans, domestic animals and triatomine vectors. Differences in EKG findings by *T*. *cruzi* serostatus were evaluated by chi-squared test. Among humans, differences in the frequency and distribution of demographic and household level variables by *T*. *cruzi* serostatus were evaluated by chi-squared test or nonparametric rank tests such as Wilcoxon ranksum. Vector count data was modeled using a negative binomial regression model to compare collections across communities. Adobe-housing material was used as the predictor of excess zeroes. A Vuong test was used to determine whether a zero-inflated negative binomial regression model was a better fit than a negative binomial regression model. Akaike’s information criterion (AIC) was used to determine that the zero-inflated negative binomial model was a better fit than the zero-inflated Poisson model. Using the model coefficient, an expected difference in vector count relative to the baseline community was calculated.

Through univariate analysis, odds ratios were estimated for the association of demographic variables (age and sex) and household level variables (presence of one or more vector, positive vector, guinea pig, positive guinea pig, dog, positive dog, or walls made of adobe) with *T*. *cruzi* seropositivity. A mixed-effects modeling approach was used, clustered by household and using an exchangeable correlation structure and logit link. Variables that have previously been shown to have an association with the outcome of interest were initially included in a multivariable logistic mixed-effects model. Using an AICc selection process, a model was constructed that included community as a fixed-effect to adjust for heterogeneity in seropositivity between communities. Because certain combinations of variables in the model resulted in a decreased sample size, the researchers ensured that the model maintained a minimum sample size of 200 subjects. It was assumed that zero vectors were present if a house was entered for data collection and the number of vectors collected was not recorded. Cohen’s Kappa analysis was conducted to test the percent of agreement between the animal serologic diagnostic methods. Statistical tests were conducted using R 3.1.3 [16], Stata 11.2, and Stata 13 (StatCorp).

## Results

### Human Study

The census enumerated 1134 people in six communities (Table 1). Of the 1093 residents older than 2 years, 612 (56.0%) participated in the serological survey. There were more female than male participants (58.5% versus 41.5%) and participants were younger than non-participants (mean age = 27.4 versus 28.2 years).

**Table 1:** The household (HH) and resident census with survey sample sizes by community.

Community	Total Households (census)	HH Surveyed for Vectors	Total Residents (census)	Participants in Serosurvey^Ŧ^	Participants in Clinical Evaluation	Dogs in Serosurvey^Ŧ^	Guinea pigs in Serosurvey^Ŧ^
Campo Florido	106	77	368	195	118	21	207
Casa Blanca	70	53	246	131	43	29	—
La Esperanza	57	42	223	123	41	37	—
Pindoc	44	36	165	75	33	21	—
Nuevo Guayaquil	—	—	87	63	19	—	—
Rumiaco	—	—	45	25	6	—	—
Total	277	208	1134	612	260	108	207

^Ŧ^One human sample, two canine samples, and one guinea pig sample were removed from analysis due to an indeterminate result, missing age and length data respectively.

Ninety-one participants (14.9%, 95% CI: 12.2–18.0%) had positive results by at least two serological assays. One participant had inconclusive results by both ELISAs and negative results by TESA-blot. His infection status therefore remained unresolved and his data were excluded from further analysis. The total study population was therefore 611 (S1 Table). Females were more likely to have *T*. *cruzi* infection than males (16.2% versus 13.0%). The seropositive population was older than those without infection (mean age 37.8 versus 25.6 years old).

**Fig 2:**
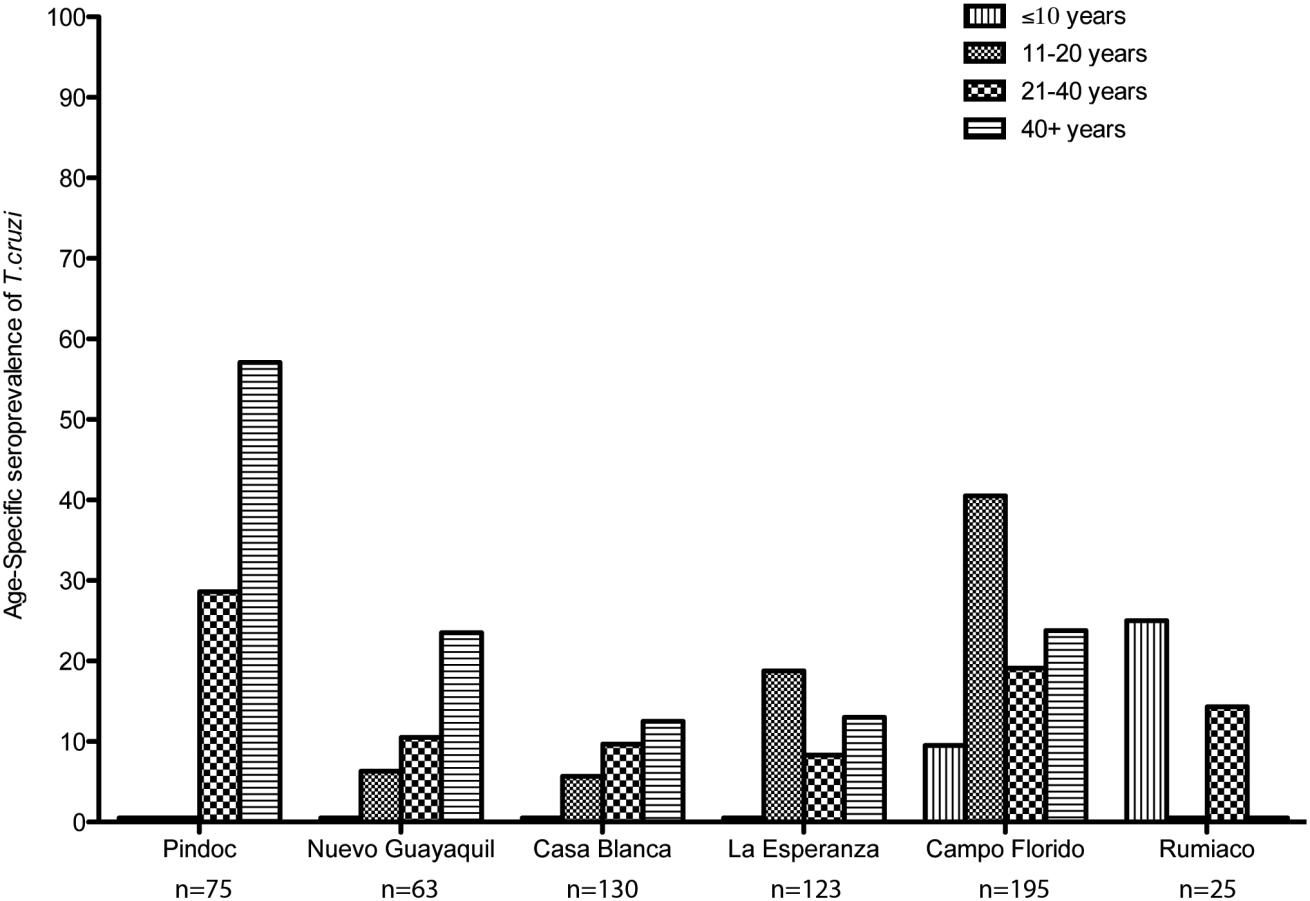
A bar graph representing seroprevalence of *T*. *cruzi* infection for each age category by community. Age-specific patterns of seroprevalence by community. In the communities of Campo Florido and Rumiaco, seroprevalence in children is notably high. This is contrary to the trend of cumulative incidence in older people correlating with a higher seroprevalence, as seen in the other communities.

Overall and age-specific seroprevalence varied across the six communities (Table 2). In Pindoc, Nuevo Guayaquil and Casa Blanca seroprevalence increased with age. However, this trend was not seen in La Esperanza, Campo Florido, and Rumiaco (Fig 2 and Table 2). Among participants <15 years old seroprevalence differed significantly between communities (ANOVA p < 0.02), with a particularly high seroprevalence in Campo Florido (17.2%, 95% CI: 9.6–24.7%).

**Table 2:** Summary of age specific seropositive study participants by community.

Community (years)	Age Category	N	*T*. *cruzi* seropositive n (%)	95% Confidence Interval
*Pindoc*		75	20 (26.7%)	17.1–38.1%
*<10*		14	0	0–23.2%
*11–20*		19	0	0–17.7%
*21–40*		14	4 (28.6%)	8.4–58.1%
*>40*		28	16 (57.1%)	37.2–75.5%
*Nuevo Guayaquil*		63	7 (11.1%)	4.6–21.6%
*<10*		11	0	0–28.5%
*11–20*		16	1 (6.3%)	0.2–30.2%
*21–40*		19	2 (10.5%)	1.3–33.1%
*>40*		17	4 (23.5%)	6.8–49.9%
*Casa Blanca*		130	10 (7.7%)	3.8–13.7%
*<10*		24	0	0–14.3%
*11–20*		35	2 (5.7%)	0.70–19.2%
*21–40*		31	3 (9.7%)	2.0–25.8%
*>40*		40	5 (12.5%)	4.2–26.8%
*La Esperanza*		123	12 (9.8%)	5.1–16.4%
*<10*		32	0	0–10.9%
*11–20*		32	6 (18.8%)	7.2–36.4%
*21–40*		36	3 (8.3%)	1.8–22.5%
*>40*		23	3 (13.0%)	2.8–33.6%
*Campo Florido*		195	40 (20.5%)	15.0–26.9%
*<10*		74	7 (9.5%)	3.9–18.5%
*11–20*		37	15 (40.5%)	24.8–57.9%
*21–40*		42	8 (19.1%)	8.6–34.1%
*>40*		42	10 (23.8%)	12.1–39.5%
*Rumiaco*		25	2 (8.0%)	1.0–26.0%
*<10*		4	1 (25.0%)	0.6–80.1%
*11–20*		8	0	0–36.9%
*21–40*		7	1 (14.3%)	0.4–57.9%
*>40*		6	0	0–45.9%

### Electrocardiogram Study

Ninety *T*. *cruzi* infected and 170 uninfected participants underwent EKGs. Both adults and children >2yo enrolled in the EKG survey, and there were more female than male matched groups (S2 Table). RBBB was rare, yet it was diagnosed in 2/90 seropositive participants and none of the 170 seronegative controls. Evaluation of the aforementioned Chagas associated EKG abnormalities showed no significant difference between seropositive and seronegative participants (4.4% of the seropositives had at least one of the EKG abnormalities versus 1.2% of seronegatives) (S3 Table).

### Entomological Household Study

Vector searches were conducted in 208 (75.1%) of the 277 houses in four communities. The search of these 208 houses was comprised of 1130 spaces: 858 rooms and 272 animal enclosures. A majority of rooms (551/858) were made of adobe (64.2%, 95% CI: 60.9–67.4%). Other less common room construction materials included brick, stone, plaster, wood, branches, and reed. The majority of roofs (560/858) were made of calamina, a corrugated roofing material of metal or plastic (65.3%, 95% CI: 62.0–68.5%). Other less common roof materials included wood or reed. Approximately half of the animal enclosures were outside of the household (128/272) and categorized as peridomestic (47.1%, 95% CI: 41.0–53.2%). Animal enclosures were most frequently made of adobe (113/272) and/or wood (100/272) (41.5%, 95% CI: 35.6–47.7%; and 36.8%, 95% CI: 31.0–42.8% respectively).

Owned domestic animals included guinea pigs, dogs, cats, chickens, turkeys, geese, ducks, pigs, sheep and cows. Some of these animals were classified as intradomiciliary and others as peridomiciliary. The most common intradomiciliary animals were guinea pigs (range 0–42) with at least one residing in 99 households (48.1%, 95% CI: 41.0–55.1%). The most common peridomicilliary animals were chickens (range 0–93), dogs (range 0–6) and pigs (range 0–11) with at least one owned by 113 (74.3%, 95% CI: 66.7–81.1%), 82 (53.9%, 95% CI: 45.7–62.0), and 76 (50.0%, 95% CI: 41.8–58.2%) households respectively.

All vectors collected were identified as one species: *Panstrongylus lignarius*. Eighty-three houses (39.9%, 95% CI: 33.2–46.9%) were infested, and 31 houses (14.9%, 95% CI: 10.4–20.5%) had at least one *T*. *cruzi*-infected vector. Triatomines were more commonly found in rooms than animal enclosures, 105/858 rooms (12.2%, 95% CI: 10.1–14.6%) and 11/272 animal enclosures (4.0%, 95% CI: 2.0–7.1%) had at least one vector present. Triatomines were collected in kitchens, eating rooms, bedrooms, empty rooms, and storage rooms, however, of the 116 spaces where triatomines were found, 59/116 (50.9%, 95% CI: 41.4–60.2%) were bedrooms and 39/116 (33.6%, 95% CI: 25.1–43.0%) were kitchens. All five nymphal stages and both sexes were found in both rooms and animal enclosures, demonstrating colonization (Table 3).

**Table 3:** Distribution of triatomine sex and developmental stage.

	Triatomine Sex		Triatomine Developmental Stage				
	Male	Female	1	2	3	4	5
Collected	283	266	167	230	390	388	289
Intestinal Contents Evaluated	279	263	—	149	333	315	286
Positive for *T*. *cruzi*	65	72	—	2	12	68	96

**Fig 3:**
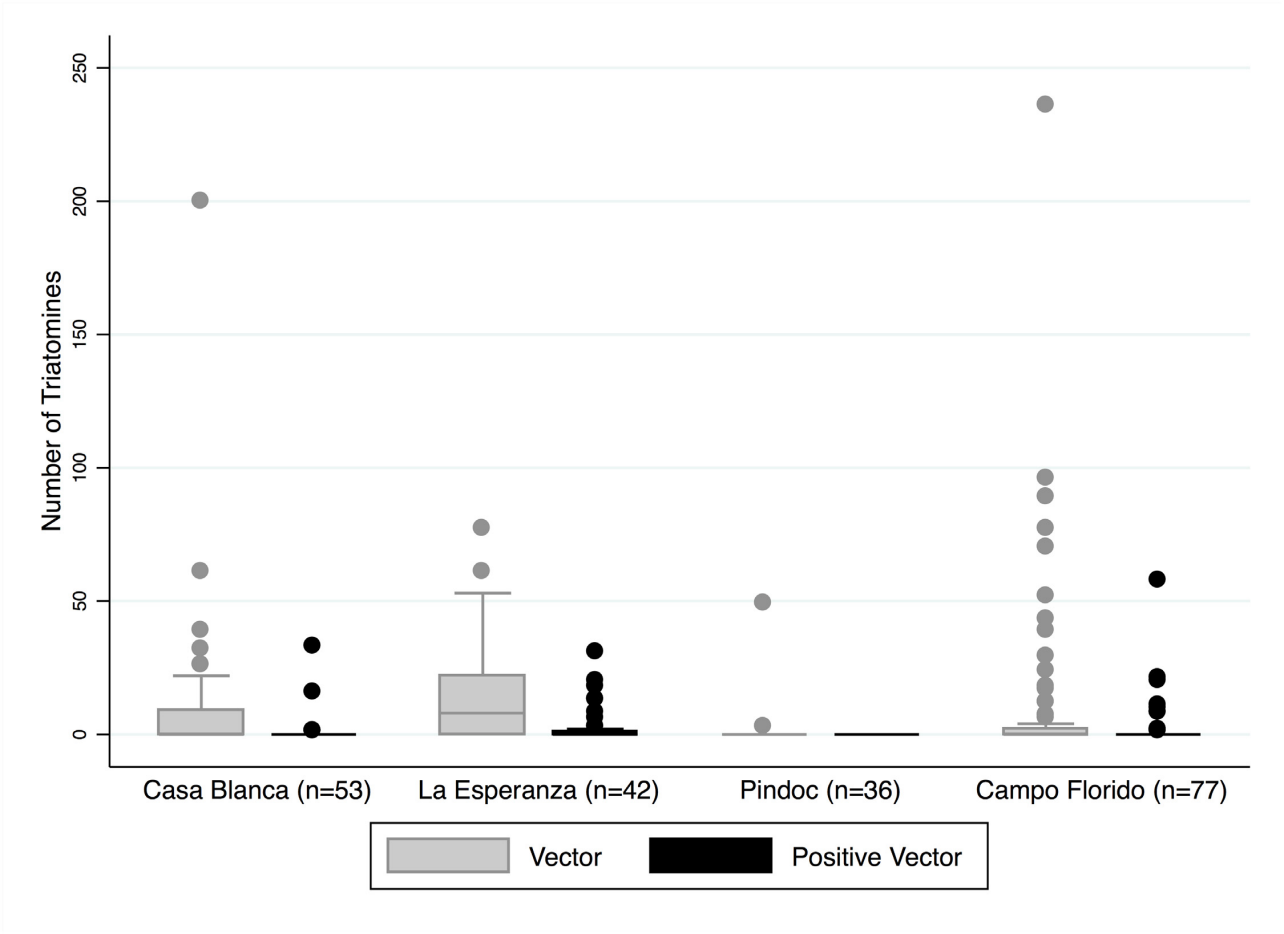
Box plots illustrating the distribution of total triatomines and *T*. *cruzi* positive triatomines by community. While the majority of houses had zero or few triatomines collected (overall median of 0), some outliers had over 200 collected from their home. The gray shaded box plots represent the total number of vectors per household, and the black box plots represent *T*. *cruzi* positive vectors per household.

In total, there were 1963 triatomines collected. The intestinal contents of 1625 triatomines were evaluated and 315 of those were positive for *T*. *cruzi* (19.4%, 95% CI: 17.5–21.4%). No other trypanosomatids were identified. A median of 0 and a mean of 10 triatomines were found per household (min 0, max 236). A zero-inflated negative binomial (ZINB) regression model examining the total household number of triatomines showed that Pindoc was significantly different from the other three communities. Pindoc had a coefficient of -1.66 (95% CI: -2.9–-0.4, z = -2.64, p<0.01), and an expected vector count of 0.19 relative to the reference community of Casa Blanca. The estimated household numbers of triatomines in La Esperanza and Campo Florido were not significantly different from Casa Blanca. A similar ZINB regression model was run examining the total household number of *T*. *cruzi* positive triatomines. Pindoc, the community where no positive vectors were captured, was found to be different from the reference community, yet there was no difference in the estimated density of positive vectors in La Esperanza and Campo Florido compared to Casa Blanca (Fig 3). The number of infected vectors showed positive correlations with the number of *T*. *cruzi*-infected dogs overall and in Campo Florido (ρ = 0.31, p<0.02; and ρ = 0.72, p < 0.01 respectively). There was a similar positive correlation in *T*. *cruzi*-infected guinea pigs (ρ = 0.84, p <0.01).

### Domestic Animal Study

The serological survey included 108 dogs (75.5%) and 207 guinea pigs (43.9%). Two dogs and one guinea pig were removed from the study due to missing age and size data, respectively. Study dogs had a mean age of 1.9 years (min 1 mo, max 15 yr) and guinea pig average length was 25.5 cm (min 20 cm, max 32 cm).

Based on EAE ELISA results, 21 dogs (19.8%; 95% CI: 12.7–28.7%) and 7 guinea pigs (3.4%; 95% CI: 1.4–6.9%) were positive for *T*. *cruzi* antibodies. There was a good agreement between ELISA and TESA-blot assays in canines (K = 0.66, 90.3% agreement, p < 0.01) and in guinea pigs (K = 0.76, 90.3% agreement p < 0.01).

### Univariate and Multivariate Modeling

In univariate analyses, risk factors for *T*. *cruzi* infection included older age and presence of infected triatomines in the house (Table 4). Owning a *T*. *cruzi* positive guinea pig showed borderline significance as a risk factor. In the multivariable model, only the presence of *T*. *cruzi* infected triatomines remained statistically significant once adjusted for community (p<0.01). People from 155/208 households in the entomological survey also participated in the human serosurvey and only these participants with corresponding household data were included in the multivariable model (477/611). Consequently, the final multivariable model included 477 observations from 155 households (Table 5). A typical individual in a given community had 6.1 greater odds of testing positive for *T*. *cruzi* when living in the presence of *T*. *cruzi* infected triatomines compared to a typical individual in the same community without positive infestation (95% CI: 1.6–22.6).

**Table 4:** Univariate analysis, risk factors for *T*. *cruzi* positive serology in rural communities of Cajamarca, Peru.

	Univariate Analysis		
Risk Factor	*T*. *cruzi* Positive Subjects/N^1^	OR^2^ (CI 95%)	p-value
**Age**			
*<20yo* ^§^	32/306	—	—
*21-40yo*	19/144	1.9 (1.0–4.8)	0.06
*41-60yo*	19/100	2.7 (1.4–8.0)	0.01
*>60yo*	21/61	8.5 (5.0–45.8)	0.01
**Sex**			
*Female*	58/611	1.4 (0.8–2.5)	0.27
**Vector**			
*Vector in house*	30/477	1.0 (0.4–2.8)	0.96
*Positive vector in house*	19/477	3.7 (1.1–12.1)	0.03
**Animals**			
*Owned Guinea Pigs*	49/464	1.3 (0.4–4.0)	0.63
*Owned Positive Guinea Pigs*	4/103	189.6 (1.0–3.6x10^3^)	0.05
*Owned Dogs*	38/464	1.6 (0.6–4.3)	0.37
*Owned Positive Dogs*	7/173	3.0 (0.4–22.4)	0.29
**Housing Material**			
*House made with Adobe walls*	61/464	0.4 (0.1–1.9)	0.25

^1^ T. cruzi positive human participants that are also described by the covariate category on the left

N = Human serosurvey participants

^2^OR, odds ratio; CI, confidence interval

^§^Reference category

**Table 5:** Multivariate analysis for *T*. *cruzi* positive serology in people of rural communities in Cajamarca, Peru.

		Multivariate Analysis	
		OR (CI 95%)	p-value
**Positive vector in house**		6.1 (1.6–22.6)	0.01
**Community**			
	*Casa Blanca* ^§^	—	—
	*La Esperanza*	1.2 (0.2–5.5)	0.84
	*Pindoc*	13.3 (2.6–69.2)	0.01
	*Campo Florido*	3.4 (0.9–13.0)	0.08

^*§*^Reference category

## Discussion

Our data show that this often-overlooked region in northern Peru has a significant Chagas disease burden and warrants additional investigation and control measures. Although Chagas disease has been documented within the range of *P*. *lignarius* in northern Peru [17], very few studies to date have examined the extent of *T*. *cruzi* infection in humans and animals and its relationship to this vector. Evidence shows a high prevalence of *T*. *cruzi* infection, 14.9%, in human residents of these six rural communities in northern Peru. Human seroprevalence in this region had previously been reported between 1–5% [7,18–21]. In southern Peru, the human seroprevalence of *T*. *cruzi* has been documented at levels ranging from 1.4 to 13.4% in urban, periurban and rural sites [22–28]. This study illustrates that secondary vector species, such as *P*. *lignarius*, play an important role in the transmission of *T*. *cruzi* and are responsible for a significant burden of Chagas disease.

Like other studies in endemic areas, our serological survey showed an increase in human seroprevalence with age [22]. Since infection is lifelong, in the absence of effective treatment, this pattern represents cumulative incidence over the residents’ lifetimes. An unusual pattern was seen in Campo Florido, Pindoc and La Esperanza. In Campo Florido in particular, the seroprevalence in children and adolescents was notably elevated, as high as 40.5% (95% CI: 24.8–57.9%) between 11 and 20 year olds. This finding does not appear to be an aberration due to small sample size, as more than 100 residents 20 or younger were tested. Rather, it appears to show both recent transmission and possibly higher risk of exposure in younger individuals. A similar pattern was seen in communities on the outskirts of Arequipa, where a mathematical model estimated that transmission began less than 20 years earlier [22,25].

One explanation for apparent recent transmission in Campo Florido is that this community never received the household insecticide application that occurred in the other five communities. According to regional governmental documentation and communications with community leaders, household residual insecticide application was carried out in Casa Blanca, La Esperanza, Pindoc, Rumiaco and Nuevo Guayaquil to reduce malaria and Bartonellosis, two vector-borne diseases that affect the region. Several insecticide treatments were undertaken at different times in different communities over the 10–15 years preceding the study, with the most recent applications taking place in Cutervo Province in 2007. Insecticide applications employed several synthetic pyrethroid compounds and may have sufficiently reduced triatomine populations to interrupt transmission over recent years. Triatomine reinfestation post spraying is the likely reason that vector density modeling showed no difference in the prevalence of household triatomines or *T*. *cruzi* infected triatomines in Campo Florido or La Esperanza compared to Casa Blanca.

Clinically, the progression to cardiac disease is the most important determinant of prognosis in patients with Chagas disease [11]. In this study, the conduction abnormality of a right bundle branch block, while rare, was found to have an association with *T*. *cruzi* serostatus, similar to findings across the Americas [2,11,29]. The presence of a right bundle-branch block alone has been associated with an increased risk of mortality in *T*. *cruzi* positive individuals, as high as a seven-fold increase in Maguire et al [30].

Despite considerable research to understand domestic animals’ roles in maintaining and augmenting *T*. *cruzi* infection, domestic species’ infection rates have great geographic variability and many questions still remain [20,31–36]. Domestic dogs are believed to be important reservoirs of the parasite, however, depending on local circumstances, dog ownership may or may not increase risk of infection [37–41]. Data from our study does not implicate dog ownership for increasing *T*. *cruzi* risk for their owners. These dogs may serve as parasite reservoirs post-insecticide spraying, however, and may contribute to the reestablishment of *T*. *cruzi* in vector populations. Serial sampling of canine serology with concurrent entomologic data before, during and after insecticide treatments may give insight into their roles as reservoirs.

Guinea pigs have historically been considered as potential *T*. *cruzi* reservoirs [7,20,36,42]; yet, evidence from this study does not implicate guinea pig ownership alone as a risk factor of human infection. Serological testing, however, may not be a reliable diagnostic in guinea pigs. Castro-Sesquen et al illustrate a slow rise of guinea pig immunoglobulin, which is only consistently detectable 40 days post *T*. *cruzi* inoculation. Considering the short life span of a domesticated guinea pig (they are commonly slaughtered for food by 3 months of age), there exists only a narrow time window when antibody levels can be sufficiently detectable even if infection occurred at a very young age [43].

Sixteen triatomine species have been reported in northern Peru, nine of which are thought to have potential to be significant vectors for *T*. *cruzi* [7,44,45]. While the majority of Amazonian triatomines are reported to be sylvatic [46], three species in northern Peru are known to be synanthropic, meaning ecologically associated with humans: *Panstronylus lignarius*, *Rhodnius ecuadoriensis*, and *Triatoma dimidata* [7]. Only one species was identified in our survey, *Panstrongylus lignarius* (syn. *P*. *herreri*) [6]. This vector has previously been called a 'domestic pest' in the Marañon River Valley [18], but has also been documented as occupying niches in sylvatic ecotopes such as bird nests in Ecuador [5]. The species *Triatoma carrioni*, *Rhodnius ecuadoriensis*, and *Panstrongylus geniculatus*, which have also been documented in Cutervo Province, were not found in this study [7]. *Triatoma infestans*, the principal vector of southern Peru responsible for transmission of *T*. *cruzi*, has never been documented north of Lima and its surrounding communities [7]. In our entomological survey, *Panstrongylus lignarius* vectors in all five nymphal stages as well as adults were found, providing evidence that a complete life cycle within domestic and peridomestic habitats is possible. In Peru, the role of extradomiciliary triatomines in *T*. *cruzi* transmission remains poorly described, though is likely similar to that in geographically proximate regions of Ecuador [47]. For vector species that are capable of inhabiting both wild and domestic ecotopes, such as *P*. *lignarius*, reinfestation after insecticide treatment is expected and long-lasting surveillance and focal control may be necessary to permanently halt transmission.

There are several limitations to our study. The serological analyses of humans and domestic animals are not directly comparable, as different sampling and diagnostic strategies were employed. The criteria for *T*. *cruzi* positivity in the human serosurvey was determined by a minimum of two out of three positive assays, whereas positivity in the domestic animal serosurveys was determined by the outcome of one ELISA assay. While there is a potential for serological misclassification in both the human and animal surveys, the misclassification rate in the human serosurvey is low on account of the three assay approach. Since vector-borne transmission was the primary focus of this study, children <2yo were excluded from the study, and consequently the role of congenital transmission was not examined. The low prevalence of infection among guinea pigs might suggest they are less relevant to *T*. *cruzi* transmission than dogs and other hosts. However, the life history of guinea pigs raised for consumption in Peru, and the time period of development of their immunological response to *T*. *cruzi* infection may obscure the interpretation of our serological tests. The prevalence of triatomine vectors and the prevalence of *T*. *cruzi* in this vector population are likely conservative estimates. The flushing out method (one person-hour) has a moderate sensitivity (76%) but has the potential to be higher in areas with higher vector density [48,49]. The timed search approach to vector detection could have been improved with the use of traps. For parasite detection, diagnostic sensitivity for *T*. *cruzi* can vary according to vector species [50,51]. While limited diagnostic information exists for the sensitivity in *Panstrongylus* species specifically, in other genera, molecular techniques can offer greater sensitivity [52,53]. Lastly, it is difficult to ascertain temporal sequence of transmission between domestic animals, vectors and humans in a cross sectional survey.

The prevalence of *T*. *cruzi* infection identified in these six communities of Cutervo Province, is equal to or higher than levels documented elsewhere in Peru, yet this region has few control measures in place, none of which targets *T*. *cruzi* and its vectors specifically. Furthermore, notably high *T*. *cruzi* seroprevalence was detected in the children and adolescents of Campo Florido. We also documented cardiac abnormalities in *T*. *cruzi* seropositive participants illustrating the potential health impacts of this protozoan to the people it infects. Prevention of Chagas related morbidity and mortality in this region may be possible with greater attention to *T*. *cruzi* infection, its vectors, and public health control strategies.

## Supporting Information

S1 Table Human serology by three different diagnostic tests, with corresponding *T*. *cruzi* infection status. (DOCX)

S2 Table Age and sex distribution of T. cruzi positive and matched *T*. *cruzi* negative groups in EKG survey. (DOCX)

S3 Table EKG abnormalities consistent with Chagas cardiomyopathy and *T*. *cruzi* serostatus (N = 260). (DOCX)

S1 Checklist STROBE checklist. (DOC)
