# Correction: Prevalence and Transmission of *Trypanosoma cruzi* in People of Rural Communities of the High Jungle of Northern Peru

**DOI:** 10.1371/journal.pntd.0003910

**Published:** 2015-07-06

**Authors:** Karen A. Alroy, Christine Huang, Robert H. Gilman, Victor R. Quispe-Machaca, Morgan A. Marks, Jenny Ancca-Juarez, Miranda Hillyard, Manuela Verastegui, Gerardo Sanchez, Lilia Cabrera, Elisa Vidal, Erica M. W. Billig, Vitaliano A. Cama, César Náquira, Caryn Bern, Michael Z. Levy, Working Group on Chagas Disease in Peru

There are several errors in this article. In Table 2, the age ranges are incorrectly listed under Community in column 1. The age ranges should be listed under Age Category in column 2. Please see the corrected Table 2 here.

There is an error in affiliation 4 for authors Robert H. Gilman, Victor R. Quispe-Machaca, Jenny Ancca-Juarez, Manuela Verastegui, Gerardo Sanchez, Lilia Cabrera, César Náquira, Michael Z. Levy, and Working Group on Chagas Disease in Peru. Affiliation 4 should be: Faculty of Science and Philosophy Alberto Cazorla Talleri, University Peruana Cayetano Heredia, Urbanización Ingeniería, Lima, Peru.

In Table 4, footnote 1 has a typo. *T*.*cruzi* should be italicized.

**Table 2:** Summary of age specific seropositive study participants by community.

Community (years)	Age Category	N	*T*. *cruzi* seropositiven (%)	95% Confidence Interval
*Pindoc*		75	20 (26.7%)	17.1–38.1%
	*<10*	14	0	0–23.2%
	*11–20*	19	0	0–17.7%
	*21–40*	14	4 (28.6%)	8.4–58.1%
	*>40*	28	16 (57.1%)	37.2–75.5%
*Nuevo Guayaquil*		63	7 (11.1%)	4.6–21.6%
	*<10*	11	0	0–28.5%
	*11–20*	16	1 (6.3%)	0.2–30.2%
	*21–40*	19	2 (10.5%)	1.3–33.1%
	*>40*	17	4 (23.5%)	6.8–49.9%
*Casa Blanca*		130	10 (7.7%)	3.8–13.7%
	*<10*	24	0	0–14.3%
	*11–20*	35	2 (5.7%)	0.70–19.2%
	*21–40*	31	3 (9.7%)	2.0–25.8%
	*>40*	40	5 (12.5%)	4.2–26.8%
*La Esperanza*		123	12 (9.8%)	5.1–16.4%
	*<10*	32	0	0–10.9%
	*11–20*	32	6 (18.8%)	7.2–36.4%
	*21–40*	36	3 (8.3%)	1.8–22.5%
	*>40*	23	3 (13.0%)	2.8–33.6%
*Campo Florido*		195	40 (20.5%)	15.0–26.9%
	*<10*	74	7 (9.5%)	3.9–18.5%
	*11–20*	37	15 (40.5%)	24.8–57.9%
	*21–40*	42	8 (19.1%)	8.6–34.1%
	*>40*	42	10 (23.8%)	12.1–39.5%
*Rumiaco*		25	2 (8.0%)	1.0–26.0%
	*<10*	4	1 (25.0%)	0.6–80.1%
	*11–20*	8	0	0–36.9%
	*21–40*	7	1 (14.3%)	0.4–57.9%
	*>40*	6	0	0–45.9%
