# Supplementary material for: Prevalence and Transmission of Trypanosoma cruzi in People of Rural Communities of the High Jungle of Northern Peru
Source: PLoS Negl Trop Dis. 2015 May 22;9(5):e0003779. doi: 10.1371/journal.pntd.0003779 (PMC4441511; doi:10.1371/journal.pntd.0003779)
Supplement: S1 Table — (DOCX) [file pntd.0003779.s001.docx]

| **S1 Table.** Human serology by three different diagnostic tests, with corresponding *T. cruzi* infection status. | | | | |
| --- | --- | --- | --- | --- |
| **Lysate ELISA (Chagatek)** | **Recombinant ELISA (Wiener)** | **Western Blot (TESAblot)** | **N** | ***T. cruzi* Infection Status** |
| NEG | NEG | NEG | 492 | NEG |
| POS | POS | POS | 86 | POS |
| NEG | POS | NEG | 25 | NEG |
| POS | POS | NEG | 3 | POS |
| POS | NEG | NEG | 2 | NEG |
| NEG | IND | NEG | 1 | NEG |
| NEG | POS | POS | 1 | POS |
| POS | POS | IND | 1 | POS |
| IND | IND | NEG | 1 | IND |
| NEG = negative, POS = positive, IND =indeterminate due to multiple discordant results | | | | |
