# Supplementary material for: Prevalence and Transmission of Trypanosoma cruzi in People of Rural Communities of the High Jungle of Northern Peru
Source: PLoS Negl Trop Dis. 2015 May 22;9(5):e0003779. doi: 10.1371/journal.pntd.0003779 (PMC4441511; doi:10.1371/journal.pntd.0003779)
Supplement: S2 Table — (DOCX) [file pntd.0003779.s002.docx]

| **S2 Table.** Age and sex distribution of *T. cruzi* positive and matched *T. cruzi* negative groups in EKG survey | | | |
| --- | --- | --- | --- |
| **Age Category** | **Male** | **Female** | **total** |
| *<10yo* | 2 | 3 | 5 |
| *11-20yo* | 9 | 16 | 25 |
| *21-40yo* | 6 | 14 | 20 |
| *41-59yo* | 6 | 13 | 19 |
| *>60yo* | 8 | 13 | 21 |
| **total** | 31 | 59 | 90 |
