# Supplementary material for: Prevalence and Transmission of Trypanosoma cruzi in People of Rural Communities of the High Jungle of Northern Peru
Source: PLoS Negl Trop Dis. 2015 May 22;9(5):e0003779. doi: 10.1371/journal.pntd.0003779 (PMC4441511; doi:10.1371/journal.pntd.0003779)
Supplement: S3 Table — (DOCX) [file pntd.0003779.s003.docx]

| **S3 Table.** EKG abnormalities consistent with Chagas cardiomyopathy and *T. cruzi* serostatus (N=260). | | | |
| --- | --- | --- | --- |
| **EKG abnormalities** | |  |  |
|  | {Subject ID Age Sex *T. cruzi* status} | |  |
|  |  | ***T. cruzi* status** | |
|  |  | **POS (n=90)** | **NEG (n=170)** |
| ***Rhythm abnormalities*** | | 0 | 0 |
| Atrial fibrillation/flutter | | 0 | 0 |
| Junctional | | 0 | 0 |
| Ventricular tachycardia | | 0 | 0 |
| Ventricular extrasystoles | | 0 | 0 |
| Sinus node dysfunction | | 1 | 0 |
|  | {CSF006-2 51yo Female *T. cruzi* Pos} | |  |
| Sinus bradycardia (<50bpm) | | 1 | 1 |
|  | {CSF029-1 21yo Male *T. cruzi* Neg} | |  |
|  | {CSF016-1 49yo Male *T. cruzi* Pos} | |  |
| ***Conduction abnormalities*** | | 0 | 0 |
| First degree AV block | | 0 | 1 |
|  | {CCP012-2 31yo Female *T. cruzi* Neg} | |  |
| Second degree AV block | | 0 | 0 |
| Third degree AV block | | 0 | 0 |
| AV disassociation | | 0 | 0 |
| Left bundle branch block | | 0 | 0 |
| Right bundle branch block | | 2 | 0 |
|  | {CCP012-1 31yo Male *T. cruzi* Pos} | |  |
|  | {CCP019-1 82yo Male *T. cruzi* Pos} | |  |
| Left anterior fascicular block | | 0 | 0 |
| Left posterior fascicular block | | 0 | 0 |
| Trifascicular block | | 0 | 0 |
