# Supplementary material for: Prevalence and Transmission of Trypanosoma cruzi in People of Rural Communities of the High Jungle of Northern Peru
Source: PLoS Negl Trop Dis. 2015 May 22;9(5):e0003779. doi: 10.1371/journal.pntd.0003779 (PMC4441511; doi:10.1371/journal.pntd.0003779)
Supplement: S1 Checklist — (DOC) [file pntd.0003779.s004.doc]

STROBE Statement—Checklist of items that should be included in reports of ***cross-sectional studies***

|  | Item No | Recommendation |
| --- | --- | --- |
| **Title and abstract** | 1 | (*a*) Indicate the study’s design with a commonly used term in the title or the abstract |
|  |  | (*b*) Provide in the abstract an informative and balanced summary of what was done and what was found |
| (a) In the Abstract Methodology section pg 2 ln 14  “A cross-sectional study was conducted to estimate the seroprevalence of *T. cruzi* infection in humans (n=611) and domestic animals [dogs (n=106) and guinea pigs (n=206)] in communities of Cutervo, Provience.”  (b) In the Abstract Methodology and conclusion sections pg 2 ln 13 – pg 3 ln 5  “Methodology:  A cross-sectional study was conducted to estimate the seroprevalence of *T. cruzi* infection in humans (n=611) and domestic animals [dogs (n=106) and guinea pigs (n=206)] in communities of Cutervo Province, Peru. Sampling and diagnostic strategies differed according to species. An entomological household study (n=208) was conducted to identify the triatomine burden and species composition, as well as the prevalence of *T. cruzi* in vectors. Electrocardiograms (EKG) were performed on a subset of participants (n=90 *T. cruzi* infected participants and 170 age and sex-matched controls). The seroprevalence of *T. cruzi* among humans, dogs, and guinea pigs was 14.9% (95% CI: 12.2 – 18.0%), 19.8% (95% CI: 12.7- 28.7%) and 3.3% (95% CI: 1.4 – 6.9%) respectively. In one community, the prevalence of *T. cruzi* infection was 17.2% (95% CI: 9.6 - 24.7%) among participants < 15 years, suggesting recent transmission. Increasing age, positive triatomines in a participant's house, and ownership of a *T. cruzi* positive guinea pig were independent correlates of *T. cruzi* infection. Only one species of triatomine was found, *Panstrongylus lignarius*, formerly *P. herreri*. Approximately forty percent (39.9%, 95% CI: 33.2 - 46.9%) of surveyed households were infested with this vector and 14.9% (95% CI: 10.4 - 20.5%) had at least one triatomine positive for *T. cruzi*. The cardiac abnormality of right bundle branch block was rare, but only identified in seropositive individuals.  Conclusions:  Our research documents a substantial prevalence of *T. cruzi* infection in Cutervo and highlights a need for greater attention and vector control efforts in northern Peru.” | | |
| Introduction | | |
| Background/rationale | 2 | Explain the scientific background and rationale for the investigation being reported |
| In the Introduction section pg 4 ln 3-8 and pg 4 ln 22 – pg 5 ln 5  “Chagas disease is endemic to poor rural regions of Central and South America and is responsible for the largest public health burden of any parasitic infection in the Western Hemisphere (1). An estimated 8 million people are infected with *T. cruzi* and millions more are at risk (2). *Trypanosoma cruzi* is carried in the gut of the triatomine vector and transmitted through the insect’s feces. While the vector-borne route predominates, oral transmission, congenital transmission and infection through organ and blood donation also occur.”  “Chagas disease is understudied in northern Peru and little is known about the epidemiology of *T. cruzi* in the region (5). *Panstrongylus lignarius* (synonymous with *Panstrongylus herreri*) (6) is known as the 'main domestic vector' of Chagas disease in northern Peru, specifically in the Marañon Valley, yet several other species have been described in northern Peru (7). We conducted a series of cross-sectional surveys in several communities of Cutervo province, in the Cajamarca region of Peru. The study aims were to (1) describe the seroprevalence of *T. cruzi* in humans, domestic dogs, and guinea pigs; (2) to describe the species and prevalence of vectors overall and with *T. cruzi;* (3) identify and characterize risk factors of *T. cruzi* infection in humans; and (4) characterize the extent and scope of cardiac abnormalities in people associated with *T. cruzi* infection.” | | |
| Objectives | 3 | State specific objectives, including any prespecified hypotheses |
| In the Introduction section pg 5 ln 1-5  “The study aims were to (1) describe the seroprevalence of *T. cruzi* in humans, domestic dogs, and guinea pigs; (2) to describe the species and prevalence of vectors overall and with *T. cruzi;* (3) identify and characterize risk factors of *T. cruzi* infection in humans; and (4) characterize the extent and scope of cardiac abnormalities in people associated with *T. cruzi* infection.“ | | |
| Methods | | |
| Study design | 4 | Present key elements of study design early in the paper |
| In the Introduction section pg 4 ln 25 – pg 5 ln 1  “We conducted a series of cross-sectional surveys in several communities of Cutervo province, in the Cajamarca region of Peru.”  In the Methods section pg 5 ln 19-22  “All six communities were included in the human serological survey and the electrocardiogram (EKG) study. A subset of four communities was sampled for domestic dog serology and for domiciliary and peridomestic vectors (Campo Florido, Casa Blanca, La Esperanza, and Pindoc) and one community (Campo Florido) was evaluated for guinea pig serology.” | | |
| Setting | 5 | Describe the setting, locations, and relevant dates, including periods of recruitment, exposure, follow-up, and data collection |
| In the Methods section pg 5 ln 9 – pg 6 ln 7  “Materials and Methods:  *Study Area and Population*  This study was conducted in December 2009 to October 2010, in Cutervo Province of Cajamarca, Peru. Cutervo is located in the Huancabamba River Valley, near the Marañon Valley of the Andes (altitude 850-1700m), which ultimately drains into the Amazon River Basin (Fig. 1). Six communities (Campo Florido, Casa Blanca, La Esperanza, Pindoc, Nuevo Guayaquil and Rumiaco) were included in the study based on government documented triatomine infestation and clinical reports of people with Chagas disease. All communities were located within an aerial distance of 15 km. They share the same ecoregion, known as the Peruvian Yungas or Selva Alta, which is characterized by neotropical forest, steep slopes and narrow valleys. Road infrastructure and access to these communities, however, was variable: Casa Blanca and La Esperanza were connected to the local highway via a gravel road; the community of Campo Florido, however, could only be reached by a poorly maintained dirt road that was impassable for several months during the rainy season. All six communities were included in the human serological survey and the electrocardiogram (EKG) study. A subset of four communities was sampled for domestic dog serology and for domiciliary and peridomestic vectors (Campo Florido, Casa Blanca, La Esperanza, and Pindoc) and one community (Campo Florido) was evaluated for guinea pig serology.  **Figure 1.** **A map and photo depicting the study region in the Peruvian high jungle.**  A map of Peru shows the region that contains the Huancabamba River Valley (A) and an inset illustrates where the communities of Casa Blanca, La Esperanza, Campo Florido, Pindoc, Rumiaco and Nuevo Guayaquil are situated (B). A photograph illustrates the town center of Campo Florido to exemplify close proximity of houses, crude housing materials, and mountainous terrain (C). Photo: Alroy  Trained study nurses recruited participants both at the local health posts during a community-wide serological testing campaign and at people’s homes during house-to-house visits.“ | | |
| Participants | 6 | (*a*) Give the eligibility criteria, and the sources and methods of selection of participants |
| In the Methods section under subheading Human Study pg 6 ln 23  “All residents of the six communities > 2 years of age were eligible to participate in the serological survey.”  In the Methods section under subheading Electrocardiogram Study pg 7 ln 15-16  “All participants of the serological survey were invited to the EKG study at the time of the serological survey recruitment.”  In the Methods section under subheading Entomological Household Study pg 8 ln 7-9  “With household member consent, two trained entomologic collectors, aided by a tetramethrin flushing-out agent (Sapolio, Mata Moscas ©), searched domestic and peridomiciliary habitats including domestic animal enclosures for a total of one half-hour (one person-hour).”  In the Methods section under subheading Domestic Animal Study pg 8 ln 22-25  “Canine age was reported by owners, and guinea pig age was approximated based on measured body length. Canine and guinea pig blood samples were collected by a veterinarian or trained phlebotomist, and, stray, pregnant, notably sick, and/or juvenile animals (dogs <1 mo, and guinea pigs < 20 cm in length) were not sampled.” | | |
| Variables | 7 | Clearly define all outcomes, exposures, predictors, potential confounders, and effect modifiers. Give diagnostic criteria, if applicable |
| In the Methods section under subheading Human Study pg 6 ln 24 and pg 7 ln 2-7.  “The age and sex of both survey participants and non-participants were recorded.”  “All human serum specimens were tested by three assays: the Chagatek *T. cruzi* lysate ELISA (bioMerieux, Marcy l’Etoile, France), the Wiener Recombinant ELISA (Wiener, Rosario Argentina), and the trypomastigote excreted-secreted antigen (TESA) immunoblot (9). *T. cruzi* infection in humans was considered confirmed if two or more tests yielded positive results (10). Specimens with one or no tests positive were considered seronegative.”  In the Methods section under subheading Electrocardiogram Study pg 7 ln 16-18 and pg 7 ln 22- pg 8 ln 3.  “Controls were matched based on age and gender. A majority of infected individuals (80) were matched with two negative controls, and the remaining individuals (10) were matched with one.”    “An EKG was considered to have abnormalities consistent with Chagas cardiomyopathy if one or more of the following were present: atrial fibrillation/flutter, junctional rhythm, ventricular tachycardia (sustained or non-sustained), ventricular extrasystoles (multiform, paired, or salvos), sinus node dysfunction, sinus bradycardia (<50bpm), second degree AV block (type I or type II), third degree AV block, AV disassociation, left or right bundle branch block (LBBB, RBBB), left anterior or left posterior fascicular block, or trifascicular block (2,11,12). Incomplete RBBB was not considered consistent with Chagas cardiomyopathy.“  In the Methods section under subheading Entomological Household Study pg 8 ln 11-16.  “Vector species was determined based on morphology. The species, quantity, sex and life stage of triatomine vectors was documented. Due to the specimen quality once the triatomines arrived at the field laboratory, not all of the collected triatomines were evaluated for sex, development stage, and intestinal contents. Second through fifth instar triatomines were evaluated for trypanosomatids. For each household the wall and roof construction material were documented; data on the total number and type of domestic animals were reported by the household representative.”  In the Methods section under subheading Domestic Animal Study pg 9 ln 1-7.  “Transport and processing were identical to that of human blood samples, however, domestic animal serostatus was determined based on an enzyme-linked immunosorbent assay (ELISA). At LID-UPCH, the domestic animal sera were tested for the presence of anti *T. cruzi* antibodies by epimastigote alkaline extract (EAE) ELISA using Arequipa strain epimastigote extracts (2.5 ug/ mL) (15). Each plate contained seven negative and one positive control. The positive control consisted of sera from either a Y strain experimentally infected guinea pig or from an Arequipa strain naturally infected dog. The sample was positive if the OD was greater than three standard deviations above the mean plate OD.” | | |
| Data sources/ measurement | 8* | For each variable of interest, give sources of data and details of methods of assessment (measurement). Describe comparability of assessment methods if there is more than one group |
| For the following variables of interest the sources of data and details of assessment method are described below.  **Human Study:**  Human demography (sex and age) pg 6 ln 24.  “The age and sex of both survey participants and non-participants were recorded.”  Human *T. cruzi* serostatus pg 6 ln 24-25 and pg 7 ln 2-7.  “Blood samples were collected from each participant, stored at 4°C and were transported on the same day to the field laboratory.”  “All human serum specimens were tested by three assays: the Chagatek *T. cruzi* lysate ELISA (bioMerieux, Marcy l’Etoile, France), the Wiener Recombinant ELISA (Wiener, Rosario Argentina), and the trypomastigote excreted-secreted antigen (TESA) immunoblot (9). *T. cruzi* infection in humans was considered confirmed if two or more tests yielded positive results (10). Specimens with one or no tests positive were considered seronegative.”  **Electrocardiogram Study:**  Presence of Chagas Cardiomyopathy pg 7 ln 18-22.  “At the local health posts, participants underwent a structured medical history, a non-invasive physical exam (PE) by a study physician, and a 12-lead EKG in the 30° inclined position (portable Welch Allyn CP100). Parents were encouraged to be present for their children’s examinations. The duration of PEs and EKGs ranged from 15-30 minutes and all EKG data was subsequently read and coded by a board certified cardiologist.“  **Entomological Household Study:**  Vector presence (species, quantity, sex, and life stage) pg 8 ln 7-9 and pg 8 ln 11-12.  “With household member consent, two trained entomologic collectors, aided by a tetramethrin flushing-out agent (Sapolio, Mata Moscas ©), searched domestic and peridomiciliary habitats including domestic animal enclosures for a total of one half-hour (one person-hour).”  “Vector species was determined based on morphology. The species, quantity, sex and life stage of triatomine vectors was documented.”  Vector *T. cruzi* infection status pg 8 ln 9-11 and pg 8 ln 12-15.  “Captured triatomines were stored at 4°C until processing at the field laboratory and then examined for the presence of *T. cruzi*, following standard procedures (13,14).“  “Due to the specimen quality once the triatomines arrived at the field laboratory, not all of the collected triatomines were evaluated for sex, development stage, and intestinal contents. Second through fifth instar triatomines were evaluated for trypanosomatids.  Construction materials (walls, roofs, animal enclosures) and household animals (number and type) pg 8 ln 15-16.  “For each household the wall and roof construction material were documented; data on the total number and type of domestic animals were reported by the household representative.”  **Domestic Animal Study:**  Domestic animal (dog and guinea pig) *T. cruzi* serostatus pg 8 ln 22 – pg 9 ln 2.  “Canine age was reported by owners, and guinea pig age was approximated based on measured body length. Canine and guinea pig blood samples were collected by a veterinarian or trained phlebotomist, and, stray, pregnant, notably sick, and/or juvenile animals (dogs <1 mo, and guinea pigs < 20 cm in length) were not sampled. Transport and processing were identical to that of human blood samples, however, domestic animal serostatus was determined based on an enzyme-linked immunosorbent assay (ELISA).”  **Comparability of Assessment Method:**  The difference of comparability between human and animal serological evaluation are described on pg 2 ln 16 and pg 19 ln 17-20.  “Sampling and diagnostic strategies differed according to species.”  “There are several limitations to our study. The serological analyses of humans and domestic animals are not directly comparable, as different sampling and diagnostic strategies were employed. The criteria for *T. cruzi* positivity in the human serosurvey was determined by a minimum of two out of three positive assays, where as positivity in the domestic animal serosurveys was determined by the outcome of one ELISA assay.” | | |
| Bias | 9 | Describe any efforts to address potential sources of bias |
| Multiple approaches for recruitment were utilized to try to minimize sampling bias in the human study pg 6 ln 6-7.  “Trained study nurses recruited participants both at the local health posts during a community-wide serological testing campaign and at people’s homes during house-to-house visits.“  In order to minimize observation bias in the electrocardiogram study, parents were encouraged to be present for their children’s examinations pg 7 ln 20.  “Parents were encouraged to be present for their children’s examinations.” | | |
| Study size | 10 | Explain how the study size was arrived at |
| A census of community members and households was undertaken in six communities. Human participants were recruited at both local health posts as well as at people’s homes door to door. More than half of all community members participated in the Human Study and approximately three quarters of all households participated in the Entomological Household Survey. This is described in the Methods and Results section pg 6 ln 6-8, pg 10 ln 10-11, and pg 12 ln 18.  “Trained study nurses recruited participants both at the local health posts during a community-wide serological testing campaign and at people’s homes during house-to-house visits.“  “The census enumerated 1134 people in six communities (Table 1). Of the 1093 residents older than 2 years, 612 (56.0%) participated in the serological survey.”  “Vector searches were conducted in 208 (75.1%) of the 277 houses in four communities.” | | |
| Quantitative variables | 11 | Explain how quantitative variables were handled in the analyses. If applicable, describe which groupings were chosen and why |
| In the Methods section under subheading Analysis pg 9 ln 16-19 and pg 9 ln 19-20.  “Vector count data was modeled using a Poisson regression model to compare collections across communities. A Vuong test was used to determine whether a zero-inflated Poisson regression model was a better fit, using adobe-housing material as the predictor of excess zeroes.”  “Through univariate analysis, odds ratios were estimated for the association of demographic variables (age and sex).” | | |
| Statistical methods | 12 | (*a*) Describe all statistical methods, including those used to control for confounding |
|  |  | (*b*) Describe any methods used to examine subgroups and interactions |
|  |  | (*c*) Explain how missing data were addressed |
|  |  | (*d*) If applicable, describe analytical methods taking account of sampling strategy |
|  |  | (*e*) Describe any sensitivity analyses |
| (a) In the Methods section under subheading Analysis pg 9 ln 11 – pg 10 ln 6.  “Descriptive statistics were first used to characterize the human study population and compare demographic information to the general population from which they were selected. The infection prevalence along with exact binomial 95% confidence intervals was ascertained for humans, domestic animals and triatomine vectors. Differences in EKG findings by *T. cruzi* serostatus was evaluated by chi-squared. Among humans, differences in the frequency and distribution of demographic and household level variables by *T. cruzi* serostatus were evaluated by chi-squared test or nonparametric rank tests such as Wilcoxon ranksum. Vector count data was modeled using a Poisson regression model to compare collections across communities. A Vuong test was used to determine whether a zero-inflated Poisson regression model was a better fit, using adobe-housing material as the predictor of excess zeroes. Through univariate analysis, odds ratios were estimated for the association of demographic variables (age and sex) and household level variables (presence of one or more vector, positive vector, guinea pig, positive guinea pig, dog, positive dog, or walls made of adobe) with *T. cruzi* seropositivity. A mixed-effects modeling approach was used, clustered by household and using an exchangeable correlation structure and logit link. Variables that have previously been shown to have an association with the outcome of interest were initially included in a multivariable logistic mixed-effects model. Because all initial variables were measured over different subsets of participants, a modified AICc selection process was used, accounting for maintaining a sample size greater than 200 subjects, to construct the most parsimonious model that included community as a fixed-effect to adjust for heterogeneity in seropositivity between communities. It was assumed that zero vectors were present if a house was entered for data collection and the number of vectors collected was not recorded. Cohen’s Kappa analysis was conducted to test the percent of agreement between the animal serologic diagnostic methods. Statistical tests were conducted using R, Stata 11.2, and Stata 13 (StatCorp).”  (b) See below on how modelling approach utilized clustering to address within household interactions pg 9 ln 22 – pg 10 ln 3.  “A mixed-effects modeling approach was used, clustered by household and using an exchangeable correlation structure and logit link. Variables that have previously been shown to have an association with the outcome of interest were initially included in a multivariable logistic mixed-effects model. Because all initial variables were measured over different subsets of participants, a modified AICc selection process was used, accounting for maintaining a sample size greater than 200 subjects, to construct the most parsimonious model that included community as a fixed-effect to adjust for heterogeneity in seropositivity between communities.”  (c) Missing data from the Household and Entomological Study were addressed as follows pg 10 ln 3-4.  “It was assumed that zero vectors were present if a house was entered for data collection and the number of vectors collected was not recorded.”  (d) Similar to question 12 (b) since some individuals in the Human Study lived within the same household, the research team utilized clustering in its modeling approach to account for within household interactions pg 9 ln 22 – pg 10 ln 3.  “A mixed-effects modeling approach was used, clustered by household and using an exchangeable correlation structure and logit link. Variables that have previously been shown to have an association with the outcome of interest were initially included in a multivariable logistic mixed-effects model. Because all initial variables were measured over different subsets of participants, a modified AICc selection process was used, accounting for maintaining a sample size greater than 200 subjects, to construct the most parsimonious model that included community as a fixed-effect to adjust for heterogeneity in seropositivity between communities.”  (e) While sensitivity analysis was not conducted, Cohen’s Kappa analysis was conducted pg 10 ln 4-5 to test for the level of agreement between two animal serologic diagnostic methods.  “Cohen’s Kappa analysis was conducted to test the percent of agreement between the animal serologic diagnostic methods.” | | |
| Results | | |
| Participants | 13* | (a) Report numbers of individuals at each stage of study—eg numbers potentially eligible, examined for eligibility, confirmed eligible, included in the study, completing follow-up, and analysed |
|  |  | (b) Give reasons for non-participation at each stage |
|  |  | (c) Consider use of a flow diagram |
| Census and study numbers were reported in a table format for ease of comparison. Table 1 on pg 10 ln 13 presented in the Results section. The table caption is:  “The household (HH) and resident census with survey sample sizes by community.”  The census and study numbers also described in the text in the Results section pg 10 ln 10-11, pg 12 ln 18, and pg 15 ln 2  “The census enumerated 1134 people in six communities (Table 1). Of the 1093 residents older than 2 years, 612 (56.0%) participated in the serological survey.”  “Vector searches were conducted in 208 (75.1%) of the 277 houses in four communities.“  “The serological survey included 108 dogs (75.5%) and 207 guinea pigs (43.9%).”  Participants that were removed from the Human and Domestic Animal Studies were described in the Results section on pg 10 ln 16 – pg 11 ln 1 and pg 15 ln 2-3, respectively.  “One participant had inconclusive results by both ELISAs and negative results by TESA-blot. His infection status therefore remained unresolved and his data were excluded from further analysis. The total study population was therefore 611 (Supplementary Table 1).”  “Two dogs and one guinea pig were removed from the study due to missing age and size data, respectively.” | | |
| Descriptive data | 14* | (a) Give characteristics of study participants (eg demographic, clinical, social) and information on exposures and potential confounders |
|  |  | (b) Indicate number of participants with missing data for each variable of interest |
| In the Results section characteristics on the human and animal participants are reported on pg 10 ln 11-12 and pg 15 ln 3-4.  “There were more female than male participants (58.5% versus 41.5%) and participants were younger than non-participants (mean age = 27.4 versus 28.2 years).”  “Study dogs had a mean age of 1.9 years (min 1 mo, max 15 yr) and guinea pig average length was 25.5 cm (min 20 cm, max 32 cm).”  Of the participants included in the analysis, there were no participants (human or domestic animal) that had missing data for sex or age/length. Missing data for covariates in univariate and multivariate analysis are described in Table 4 and Table 5 on pg 15 ln 18 and pg 16 ln 1, respectively. | | |
| Outcome data | 15* | Report numbers of outcome events or summary measures |
| The number of outcome events are reported in the Results section **Human Study** pg 10 ln 15-16**:**  “Ninety-one participants (14.9%, 95% CI: 12.2 – 18.0%) had positive results by at least two serological assays.”  **Electrocardiogram Study** pg 12 ln 12-13**:**  **“**RBBB was rare, yet it was diagnosed in 2/90 seropositive participants and none of the 170 seronegative controls.”  **Entomological Household Study** pg 13 ln 16-18**:**  “All vectors collected were identified as one species: *Panstrongylus lignarius*. Eighty-three houses (39.9%, 95% CI: 33.2 - 46.9%) were infested, and 31 houses (14.9%, 95% CI: 10.4 - 20.5%) had at least one *T. cruzi*-infected vector*.”*  The number and distribution of sex and development stage of Triatomines are described in Table 3 pg 14 ln 2. The table caption is:  “Distribution of triatomine sex and developmental stage.”  **Domestic Animal Study** pg 15 ln 5-6**:**  “Based on EAE ELISA results, 21 dogs (19.8%; 95% CI 12.7- 28.7%) and 7 guinea pigs (3.4%; 95% CI: 1.4 – 6.9%) were positive for *T. cruzi* antibodies.” | | |
| Main results | 16 | (*a*) Give unadjusted estimates and, if applicable, confounder-adjusted estimates and their precision (eg, 95% confidence interval). Make clear which confounders were adjusted for and why they were included |
|  |  | (*b*) Report category boundaries when continuous variables were categorized |
|  |  | (*c*) If relevant, consider translating estimates of relative risk into absolute risk for a meaningful time period |
| The main results are described in the Results section under the subheading Univariate and Multivariate Modeling pg 15 ln 11-17**:**  “In univariate analyses, risk factors for *T. cruzi* infection included older age and presence of infected triatomines in the house (Table 4). Owning a *T. cruzi* positive guinea pig showed borderline significance as a risk factor. In the multivariable model, only the presence of *T. cruzi* infected triatomines remained statistically significant once adjusted for community (p<0.01). The final multivariable model included 477 observations among 155 households (Table 5). A typical individual in a given community had 6.1 greater odds of testing positive for *T. cruzi* when living in the presence of *T. cruzi* infected triatomines compared to a typical individual in the same community without positive infestation (95% CI: 1.6 – 22.6).”  Table 4 pg 15 ln 18 and 5 pg 16 ln 1 describe “Univariate analysis, risk factors for *T. cruzi* positive serology in rural communities of Cajamarca, Peru” and “Multivariate analysis for *T. cruzi* positive serology in rural communities of Cajamarca, Peru” Respectively. Age category boundaries are described in Table 4. | | |
| Other analyses | 17 | Report other analyses done—eg analyses of subgroups and interactions, and sensitivity analyses |
| Other analyses are reported in the Results section include the following on pg 14 ln 6-13, pg 14 ln 13-16, and pg 15 ln 6-8.  “A zero-inflated Poisson (ZIP) regression model examining the total household number of triatomines showed that Campo Florido had 1.4 times the estimated vector density compared to Casa Blanca (95% CI: 1.2 – 1.5, z=5.56, p<0.01), but that the household number of triatomines in La Esperanza and Pindoc were not significantly different from Casa Blanca. A similar ZIP regression model was run examining the total household number of *T. cruzi* positive triatomines, which showed that Campo Florido had 1.5 times the estimated density of positive vectors than Casa Blanca (95% CI: 1.1 – 2.0, z=2.36, p<0.02) while La Esperanza and Pindoc were not significantly different from Casa Blanca (Fig. 3).”  “The number of infected vectors showed positive correlations with the number of *T. cruzi*-infected dogs overall and in Campo Florido (ρ = 0.31, p<0.02; and ρ = 0.72, p < 0.01 respectively). There was a similar positive correlation in *T. cruzi*-infected guinea pigs (ρ = 0.84, p <0.01).”  “There was a good agreement between ELISA and TESA-blot assays in canines (Κ=0.66, 90.3% agreement, p < 0.01) and in guinea pigs (K = 0.76, 90.32% agreement p < 0.01).” | | |
| Discussion | | |
| Key results | 18 | Summarise key results with reference to study objectives |
| The key findings from the study are summarized in the Discussion section on pages pg 16 ln 7-9, pg 18 ln 5-6, pg 18 ln 13-14, pg 18 ln 19-20 and pg 19 ln 5-6.  “Evidence shows a high prevalence of *T. cruzi* infection, 14.9%, in human residents of these six rural communities in northern Peru. Human seroprevalence in this region had previously been reported between 1-5% (7,17–20).”  “In this study, the conduction abnormality of a right bundle branch block, while rare, was found to have an association with *T. cruzi* serostatus, similar to findings across the Americas (2,11,30).”  “Data from our study does not implicate dog ownership for increasing *T. cruzi* risk for their owners.”  “Guinea pigs have historically been considered as potential *T. cruzi* reservoirs (7,19,37,43); yet, evidence from this study does not implicate guinea pig ownership alone as a risk factor of human infection.”  “Only one species was identified in our survey, *Panstrongylus lignarius* (syn. *P. herreri*) (6).” | | |
| Limitations | 19 | Discuss limitations of the study, taking into account sources of potential bias or imprecision. Discuss both direction and magnitude of any potential bias |
| The limitations of the study are described in the Discussion section on pg 19 ln 17 – pg 20 ln 10.  “There are several limitations to our study. The serological analyses of humans and domestic animals are not directly comparable, as different sampling and diagnostic strategies were employed. The criteria for *T. cruzi* positivity in the human serosurvey was determined by a minimum of two out of three positive assays, where as positivity in the domestic animal serosurveys was determined by the outcome of one ELISA assay. While there is a potential for serological misclassification in both the human and animal surveys, the misclassification rate in the human serosurvey is low on account of the three assay approach. Since vector-born transmission was the primary focus of this study, children <2yo were excluded from the study, and consequently the role of congenital transmission was not examined. The low prevalence of infection among guinea pigs might suggest they are less relevant to *T. cruzi* transmission than dogs and other hosts. However, the life history of guinea pigs raised for consumption in Peru, and the time period of development of their immunological response to *T. cruzi* infection may obscure the interpretation of our serological tests. The prevalence of triatomine vectors and the prevalence of *T. cruzi* in this vector population are likely conservative estimates. The flushing out method (one person-hour) has a moderate sensitivity (76%) but has the potential to be higher in areas with higher vector density (48,49). The timed search approach to vector detection could have been improved with the use of traps. For parasite detection, diagnostic sensitivity for *T. cruzi* can vary according to vector species (50,51). While limited diagnostic information exists for the sensitivity in *Panstrongylus* species specifically, in other genera, molecular techniques can offer greater sensitivity (52,53). Lastly, it is difficult to ascertain temporal sequence of transmission between domestic animals, vectors and humans in a cross sectional survey.” | | |
| Interpretation | 20 | Give a cautious overall interpretation of results considering objectives, limitations, multiplicity of analyses, results from similar studies, and other relevant evidence |
| The overall interpretation of the study is described at the end of the Discussion section on pg 20 ln 12-18.  “The prevalence of *T. cruzi* infection identified in these six communities of Cutervo province, is equal to or higher than levels documented elsewhere in Peru, yet this region has few control measures in place, none of which target *T. cruzi* and its vectors specifically. Furthermore, notably high *T. cruzi* seroprevalence was detected in the children and adolescents of Campo Florido. We also documented cardiac abnormalities in *T. cruzi* seropositive participants illustrating the potential health impacts of this protozoan to the people it infects. Prevention of Chagas related morbidity and mortality in this region may be possible with greater attention to *T. cruzi* infection*,* its vectors, and public health control strategies.” | | |
| Generalisability | 21 | Discuss the generalisability (external validity) of the study results |
| The generalizability of the findings from this study are described in the context of *T. cruzi* infection elsewhere in Peru and in South America in the following parts of the Discussion section pg 16 ln 7 – pg 17 ln 2, pg 17 ln 4-6, pg 18 ln 19-25 and pg 19 ln 8-15.  “Evidence shows a high prevalence of *T. cruzi* infection, 14.9%, in human residents of these six rural communities in northern Peru. Human seroprevalence in this region had previously been reported between 1-5% (7,17–20). In southern Peru, the human seroprevalence of *T. cruzi* has been documented at levels ranging from 1.4 to 13.4% in urban, periurban and rural sites (21–27).”  “Like other studies in endemic areas, our serological survey showed an increase in human seroprevalence with age (21,28). Since infection is lifelong, in the absence of effective treatment, this pattern represents cumulative incidence over the residents’ lifetimes.”  “Guinea pigs have historically been considered as potential *T. cruzi* reservoirs (7,19,37,43); yet, evidence from this study does not implicate guinea pig ownership alone as a risk factor of human infection. Serological testing, however, may not be a reliable diagnostic in guinea pigs. Castro-Sesquen et al illustrate a slow rise of guinea pig immunoglobulin, which is only consistently detectable 40 days post *T. cruzi* inoculation. Considering the short life span of a domesticated guinea pig (they are commonly slaughtered for food by 3 months of age), there exists only a narrow time window when antibody levels can be sufficiently detectable even if infection occurred at a very young age (44).“  “The species *Triatoma carrioni, Rhodnius ecuadoriensis,* and *Panstrongylus geniculatus*, which have also been documented in Cutervo Province, were not found in this study (7)*.* *Triatoma infestans,* the principal vector of southern Peru responsible for transmission of *T. cruzi*, has never been documented north of Lima and its surrounding communities (7). In our entomological survey, *Panstrongylus lignarius* vectors in all five nyphal stages as well as adults were found, suggesting that a complete life cycle within domestic and peridomestic habitats is possible. In Peru, the role of extradomiciliary triatomines in *T. cruzi* transmission remains poorly described, though is likely similar to that in geographically proximate regions of Ecuador (38).” | | |
| Other information | | |
| Funding | 22 | Give the source of funding and the role of the funders for the present study and, if applicable, for the original study on which the present article is based |

Funding information and the role of the funders was presented in the Financial Disclosure statement submitted to the Journal PloS NTD.

“Karen Alroy and Victor Quispe-Machaca were 2010-2011 scholars and Christine Huang and Miranda Hillyard were 2009-2010 scholars in the Fogarty International Clinical Research Scholars Program at Vanderbilt University (R24 TW007988 & http://www.nih.gov/), supported by the National Institutes of Health Office of the Director, Fogarty International Center, Office of AIDS Research, National Cancer Center, National Eye Institute, National Heart, Blood, and Lung Institute, National Institute of Dental & Craniofacial Research, National Institute On Drug Abuse, National Institute of Mental Health, National Institute of Allergy and Infectious Diseases Health, NIH Office of Women’s Health and Research, and the American Relief and Recovery Act.

Mike Levy was supported by a grant from the National Institute of Allergy and Infectious Diseases of the National Institutes of Health (NIH-NIAID) (5R01AI101229 & http://www.niaid.nih.gov/).

Erica Billig was supported by a training grant from the National Institutes of Allergy and Infectious Diseases of the National Institutes of Health (NIH-NIAID) (5T32AI007532-17 & http://www.niaid.nih.gov/).

The funders had no role in study design, data collection and analysis, decision to publish, or preparation of the manuscript.”

*Give information separately for exposed and unexposed groups.

**Note:** An Explanation and Elaboration article discusses each checklist item and gives methodological background and published examples of transparent reporting. The STROBE checklist is best used in conjunction with this article (freely available on the Web sites of PLoS Medicine at http://www.plosmedicine.org/, Annals of Internal Medicine at http://www.annals.org/, and Epidemiology at http://www.epidem.com/). Information on the STROBE Initiative is available at www.strobe-statement.org.
